# Transcription Factors and Plants Response to Drought Stress: Current Understanding and Future Directions

**DOI:** 10.3389/fpls.2016.01029

**Published:** 2016-07-14

**Authors:** Rohit Joshi, Shabir H. Wani, Balwant Singh, Abhishek Bohra, Zahoor A. Dar, Ajaz A. Lone, Ashwani Pareek, Sneh L. Singla-Pareek

**Affiliations:** ^1^Plant Stress Biology, International Centre for Genetic Engineering and BiotechnologyNew Delhi, India; ^2^Division of Genetics and Plant Breeding, Sher-e-Kashmir University of Agricultural Sciences and Technology of KashmirSrinagar, India; ^3^National Research Centre on Plant BiotechnologyNew Delhi, India; ^4^Crop Improvement Division, Indian Institute of Pulses ResearchKanpur, India; ^5^Dryland Agricultural Research Station, Sher-e-Kashmir University of Agricultural Sciences and Technology of KashmirBudgam, India; ^6^Stress Physiology and Molecular Biology Laboratory, School of Life Sciences, Jawaharlal Nehru UniversityNew Delhi, India

**Keywords:** drought, transcription factors, climate change, abiotic stress, crop plants

## Abstract

Increasing vulnerability of plants to a variety of stresses such as drought, salt and extreme temperatures poses a global threat to sustained growth and productivity of major crops. Of these stresses, drought represents a considerable threat to plant growth and development. In view of this, developing staple food cultivars with improved drought tolerance emerges as the most sustainable solution toward improving crop productivity in a scenario of climate change. In parallel, unraveling the genetic architecture and the targeted identification of molecular networks using modern “OMICS” analyses, that can underpin drought tolerance mechanisms, is urgently required. Importantly, integrated studies intending to elucidate complex mechanisms can bridge the gap existing in our current knowledge about drought stress tolerance in plants. It is now well established that drought tolerance is regulated by several genes, including transcription factors (TFs) that enable plants to withstand unfavorable conditions, and these remain potential genomic candidates for their wide application in crop breeding. These TFs represent the key molecular switches orchestrating the regulation of plant developmental processes in response to a variety of stresses. The current review aims to offer a deeper understanding of TFs engaged in regulating plant’s response under drought stress and to devise potential strategies to improve plant tolerance against drought.

## Introduction

The 21st century agriculture is facing a daunting challenge of attaining nearly up to 70% increase in crop productivity by 2050 (Friedrich, 2015; Joshi et al., 2016a; Wang et al., 2016b). Also, crop productivity needs to witness up to 40% increase by 2015 in view of the cultivated area being increasingly affected by stresses (Pennisi, 2008). Frequent changes in climatic conditions also affect the crop yield (such as flooding after high temperature), which led scientists to undertake research on this aspect in recent time around the world (Nakashima et al., 2014a). Among the various abiotic factors challenging crop production globally, drought stress is increasingly playing a crucial role. Drought is a meteorological term and is commonly defined as a combined interplay of reduced rainfall, decreasing ground water table, limiting water availability with rise in temperature (Singh and Laxmi, 2015; Singh B. et al., 2015). With global climatic vagaries registering escalated frequency, the global drought incidence is likely to swell beyond 20% by the end of this century, especially in Central and South American corn belt, and Central and Western Europic regions (Edmeades, 2013; Singh B. et al., 2015). Similarly in South Asia, where more than 75% of the farmers are dependent on rainfed agriculture, a damage of about US$ 84 billion is predicted owing to the global climate change (Mendelsohn, 2014). Most of the crops are susceptible to drought stress, resulting in more than 50% yield losses (Singh B. et al., 2015). In South and Southeast Asia, drought stress causes about 40% annual loss in productivity, which results in 58% of income loss (Li et al., 2015). Similarly, regional climate change models showed Mediterranean regions and Middle East among the ‘Hot-spots’ of severe drought, directly affecting crop failures and livestock death (Barlow et al., 2015; Saadi et al., 2015). Brazil, which is the second highest producer (40%) of soybean has lost greater than 20% of their productivity because of the occurrence of drought during 2003–2005 (Nakashima et al., 2014b). The uneven distribution of rainfall and ground water shortage often create drought stress conditions in the environment (Luo and Zhang, 2001), which eventually leads to an enormous decrease in the grain yield potential.

Upon exposure to drought, plants manifest multiple impairments including cell injury through reactive oxygen species (ROS) generation and increasing cellular temperature, which result in an increase in the viscosity of cellular contents, alterations in the protein–protein interactions, protein aggregation, and denaturation (Farooq et al., 2008). Cell shrinkage followed by a marked decline in cellular volume becomes evident as an instant symptom caused by dehydration. Besides, greater accumulation of solutes causes toxicity and negatively affects functioning of some enzymes, often leading to reduced photosynthesis and water use efficiency (WUE). Under prolonged dehydration, plants exhibit leaf rolling followed by wilting and bleaching that eventually result in death of the plant (Sahoo et al., 2013). Reproductive stages, i.e., flowering as well as seed development are especially sensitive to drought stress (Samarah et al., 2009a,b,c; Alqudah et al., 2010; Samarah and Alqudah, 2011). A reduction in number of grains per spike, grain filling duration, and dry matter accumulation, leading to decreased grain weight in barley kernels has already been reported earlier (Samarah et al., 2009a; Samarah and Alqudah, 2011). Recently, leaf area, leaf weight as well as leaf growth rate were also reported to hold great relevance while breeding crops grown under Mediterranean environmental conditions such as drought stress (Alqudah and Schnurbusch, 2015; Quan et al., 2016). Substantial progress has already been made to enhance crop productivity using conventional breeding methods. However, development of drought tolerant cultivars using conventional breeding approach is greatly restricted by low heritability of drought tolerance, narrow genetic variation existing in the crop’s exploitable genetic pool, complex and multigenic nature of this trait and high magnitude of environmental interactions (Pardo, 2010; Hill et al., 2013; Jha et al., 2014; Liu J.H. et al., 2014).

Considering global food security, immediate assessment of drought stress impact and innovative techniques to breed tolerant and productive cultivars under dramatically changing climate are essentially warranted (Mishra and Singh, 2010). As a means to provide crop-based answer to burgeoning issues like food insecurity and malnutrition, cereals constitute a vital role as major fraction in the diet, especially in developing nations (Bohra et al., 2015). Rice, wheat and maize are major cereal crops in the world; therefore less water availability exerts profound consequences (Joshi et al., 2011). Development of advanced genotypes with improved stress tolerance and wider adaptability is a simple, cost-effective and eco-friendly approach to cope with drought stressed scenarios (Jha et al., 2014).

Drought tolerance is an outcome of a series of molecular, cellular, and physiological processes including induction/repression of various genes that cause accumulation of various osmolytes, improved antioxidant system, reduced transpiration, inhibited shoot growth, and decreased tillering (Pareek et al., 2010). Besides, the phytohormone abscisic acid (ABA) is reported to be abundant under water-deficit conditions and this in turn causes stomata closure and induces expression of various stress-related genes (Yang et al., 2011). It has been shown that drought inducible gene expression is also governed by ABA-independent regulatory system (Aguado et al., 2014). Plant’s ability to cope with water deficit depends largely on its water status, which changes with environmental conditions (Joshi et al., 2016b). Thus, incorporating drought stress tolerance in crops becomes imperative under changing climatic conditions and it could be a promising approach to meet the global food demand (Todaka et al., 2012). In this context, unraveling the molecular mechanisms that control the perception and transduction of stress signals to initiate adaptive responses is crucial for engineering drought stress tolerance in plants (Ray et al., 2010; Sanchez et al., 2011).

**FIGURE 1:**
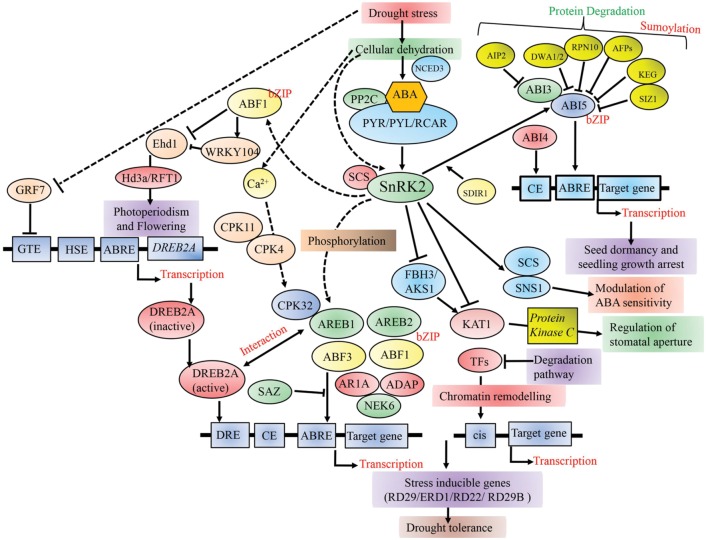
**A schematic model of the transcriptional regulation of different TFs playing key roles in cellular dehydration in plants.** PP2C-PYR/PYL/RCAR complex positively regulates AREB/ABF-SnRK2 pathway. SnRK2s further modulate other TFs downstream including AREB/ABFs, FBH3/AKS1, and SNS1 during stress as well as seed maturation. Phosphorylated AREB/ABF TFs, AREB1, AREB2, ABF3, and ABF1 bind to the promoter region of target genes and activate their expression in response to dehydration stress. GRF7 suppresses the expression of DREB2A, which is a key TF in ABA-independent gene expression. Broken lines indicate possible roles. PP2C-PYR/PYL/RCAR, pyrabactin resistance1/PYR1-like/regulatory components of ABA receptors; PP2C, protein phosphatase 2C; CE, coupling element; GTE, GRF7-targeting *cis* element; HSE, heat shock element; TFs, transcription factors.

Notable progress has been made toward this end by utilizing modern genetics and functional genomics approaches such as transcriptomics, proteomics and metabolomics and consequently, various drought stress responsive genes have been identified and characterized in crops. These key genes mainly code for proteins that have either metabolic or regulatory roles, such as those involved in detoxification, osmolyte biosynthesis, proteolysis of cellular substrates, water channel, ion transporter, heat shock protein (HSP), and late embryogenesis abundant (LEA) protein (Joshi et al., 2016b). On the other hand, the regulatory class primarily comprises of TFs (AREB, AP2/ERF, NAC, bZIP, MYC, and MYB), signaling protein kinases [mitogen activated protein kinases (MAPK), calcium-dependent protein kinases (CDPK), receptor protein kinases, ribosomal protein kinases, and transcription regulation protein kinases] and protein phosphatases (phosphoesterases and phospholipase), which synchronize signal transduction and expression of genes during stress responses (Wani et al., 2013). Various desiccation-induced genes have been characterized, including those encoding ABA biosynthetic pathway (Sah et al., 2016), osmo-protectants such as proline, trehalose, mannitol, ectoine, glycine betaine providing tolerance to cellular dehydration (Wani and Gosal, 2011; Yao et al., 2011; Varshney et al., 2011), cellular enzymes (Puckette et al., 2007), signaling proteins (Zhu, 2002), and TFs (Mochida et al., 2009; Gosal et al., 2010). Several of these regulatory genes including TFs were found to play essential roles in multiple abiotic stress responses via regulating downstream stress-responsive genes. As illustrated in **Figure 1**, TFs regulate gene expression through binding to *cis*-regulatory elements in the promoter region of different stress-related genes (Nuruzzaman et al., 2013; Franco-Zorrilla et al., 2014). Thus, genetic modification of the expression of these regulatory genes can greatly influence plant stress tolerance because they further regulate many downstream stress-responsive genes at a given time (Wang et al., 2016). Among these regulatory genes, stress-responsive TFs have gained widespread attention on account of their significant role in plant stress tolerance (Shao et al., 2015) and allelic variations were examined across Indian wild rice population by researchers in genes with respect to abiotic stresses (Singh et al., 2015a; Mishra et al., 2016). In this article, we elaborate on the key TFs that contribute toward drought stress tolerance, and their networks, cross-talks and potential utilization in crop improvement programs.

### Molecular Mechanisms Regulating Plant’s Response toward Drought Stress

The response of any crop to drought stress depends primarily on the growth stage and WUE (Pareek et al., 2010). For example, reproductive stage is considered critically susceptible to drought stress in various crops (Moumeni et al., 2015; Farooq et al., 2016). As sessile organisms, plants have evolved several defense mechanisms involving various molecular, physiological and biochemical alterations in response to low soil moisture. Visible symptoms describing drought tolerance include leaf rolling, stay green ability, epicuticular wax deposition, closing of stomata, enhanced root length leading to higher WUE, photochemical quenching, photoinhibition resistance, osmotic adjustment, and membrane stabilization at the cellular level (Khazaei et al., 2013). Leaf rolling, a foremost symptom, constitutes an initial survival mechanism against osmotic stress that can minimize canopy temperature and transpiration rate, thereby improving WUE (Singh B. et al., 2015). Thus, species maintaining higher relative water content (RWC) under osmotic stress are reported to be less susceptible to low water potential, and thus retain their growth and productivity (Joshi and Karan, 2013). It was also observed that crop growth is primarily supported by the soil moisture surrounding plant root system (Tron et al., 2015). Therefore, total root area being directly affected by the decreased soil water content deserves greater attention. It was reported that osmotic stress-induced premature differentiation caused growth inhibition of primary roots and allows outgrowth of lateral roots (Ji et al., 2014). A range of biochemical changes have also shown in response to drought stress, including higher accumulation of osmolytes, ROS, antioxidant species, and antioxidative enzymes (Kholová et al., 2011). These osmolytes are reported to have principal role in maintaining enzyme activity and membrane stability under osmotic stress (Du et al., 2014; Khattab et al., 2014; Singh B. et al., 2015).

Drought stress in plants occurs either due to poor quantity of water, i.e., little rainfall or due to insufficient quality of water, i.e., saline habitats (Buchanan et al., 2015). As a means to survive under water stress conditions, plants respond by alteration in expression of several genes and regulate them through complex transcriptional networks (Singh and Laxmi, 2015). It is therefore imperative to dissect stress regulatory mechanisms and to pinpoint the key regulators that could eventually be harnessed to breed or engineer stress tolerant plants. By analyzing plant genomes and employing modern OMICS tools, including genomics, transcriptomics and proteomics, significant progress has been made in elucidating the stress signaling pathways involved in drought stress response (Liu J.H. et al., 2014). Few of these genes associated with these transcriptional networks have been characterized in various molecular studies and have been found to be key contributors toward drought stress tolerance in transgenic plants (Todaka et al., 2015).

The signaling pathway of any abiotic stress involves certain key steps such as signal perception, transduction, responsiveness, combined with activation of physiological, and metabolic reactions (Pérez-Clemente et al., 2013; Liu J.H. et al., 2014). In this process, plant cells first perceive stress stimulus through sensors or receptors localized mostly at the cell membrane. Then, the receiver signaling molecules activate the intracellular ones through second messengers like calcium ions, inositol phosphate, ROS, cyclic nucleotides (cAMP and cGMP), sugars and nitric oxide. Subsequently, these second messengers initiate the corresponding signaling pathways to transduce the signals (Bhargava and Sawant, 2013). The phosphorylation and dephosphorylation of proteins regulated by protein kinases and phosphatases, respectively, is a significant and effective mechanism in several signal transduction pathways. For example, the MAPKs and CDPKs are well known for their role in drought stress signaling pathways (Huang et al., 2012). At the end of the phosphorylation cascade, TFs are activated or suppressed by protein kinases or phosphatases, and directly regulate the expression of an array of downstream genes by interacting with the specific *cis*-elements in their promoter region (Danquah et al., 2014). In addition, TFs themselves are regulated at the transcription level by other upstream components (Hirayama and Shinozaki, 2010) and after several modifications at the post-transcription level, such as ubiquitination and sumoylation, they form a complex regulatory network which modulates the expression of stress responsive genes, regulating various physiological and metabolic processes (Mizoi et al., 2013). In the past few decades, considerable research has been carried out toward identification and characterization of different TFs that contribute toward drought stress response.

### TFs Can Induce a Range of Stress Responsive Genes in Plants

The multifarious signaling pathways of water stress in plants consist of several proteins, i.e., TFs, enzymes, functional proteins, molecular chaperones, and metabolites (Song et al., 2013). Genetic engineering approaches to mitigate the challenge of drought stress in plants involve the overexpression of these TFs, enzymes and other metabolites (Singh B. et al., 2015). In recent years, a wide range of TF families holding relevance with drought stress response have been identified (Anbazhagan et al., 2015). During the signal transduction, TFs directly regulate the expression of the associated genes via serving as molecular switches. These TFs interact specifically with *cis*-elements located in the promoter region of the genes they regulate. In plants, a large proportion of genes in the genome (up to 10%) potentially encode TFs (Franco-Zorrilla et al., 2014), which are categorized into different gene families such as *AREB, DREB, MYB, WRKY, NAC*, and *bZIP* based on the distinct structure of their DNA-binding domain (Golldack et al., 2011; Jin et al., 2014). In *Arabidopsis* nearly 6% of the proteome is dedicated to TFs (Rayko et al., 2010). Among these TF genes, several have been reported to respond under drought stress through pathways dependent/independent of ABA. Promising results have been obtained concerning engineering of some TF genes with an aim to improve tolerance against drought stress. In this section, we describe major TF families known to be responsible for drought stress tolerance and discuss the scope for manipulating them in order to obtain transgenic plants with enhanced drought tolerance.

### AREB/ABF TFs

Several research groups have elucidated the molecular mechanism related to drought stress transcriptional networks in plants. Under osmotic stress conditions, detailed molecular analyses have found abscisic acid-responsive element binding protein (*AREB)/ABFs* (ABRE binding factor) as a major transcriptional activator modulating the expression of genes during ABA signaling (Maruyama et al., 2012). ABA-responsive gene expression is controlled by a conserved ABRE (PyACGTGG/TC) *cis*-element in its promoter region (**Figure 1**). Studies have revealed the essential role of several *ABRE* or its combinations with coupling elements (CE) during ABA-responsive genes expression (Fujita et al., 2013; Nakashima and Yamaguchi-Shinozaki, 2013). Structurally, *AREB/ABFs* consist of four SnRK2 phosphorylation sites containing conserved domains that regulate ABA-dependent gene expression (Fujita et al., 2011, 2013). It was found that vascular tissue specific cells produce ABA and transfer them to target cells (Kuromori et al., 2014). It was also demonstrated that ABA is synthesized within guard cells and vascular tissue specific cells (Bauer et al., 2013). In *Arabidopsis*, five genes are known to code for NCED (9-*cis*-epoxy carotenoid dioxygenase), a major enzyme in ABA biosynthesis and expression. Of these, *NCED3* showed higher expression under dehydration and was reported to be under the regulation of an AG-box recognition sequence located 2248 bp upstream to its transcriptional start site (Behnam et al., 2013). ABA is perceived by an ABA-bound PYL/PYR/RCARs receptor complex combined with PP2Cs, and subsequently SnRK2s is released (Miyakawa et al., 2013; Nakashima and Yamaguchi-Shinozaki, 2013). The activated SnRKs phosphorylate the downstream proteins like AREB/ABF TFs, which then bind to the ABRE *cis*-element (PyACGTGG/TC) (Umezawa et al., 2013). In the absence of ABA, PP2Cs inhibit ABA signaling pathway by dephosphorylating SnRK2s. Of the nine AREB/ABF family members, AREB1/ABF2 regulates ABA signaling during drought stress at the vegetative stage (**Figure 1**). ABA is essential for proper functioning of AREB1, and ABA-dependent phosphorylation regulates its activity (Yoshida et al., 2010). Overexpression of *AREB1* was shown to improve drought tolerance in *Arabidopsis*, rice and soybean (Oh et al., 2005; Barbosa et al., 2013; Yoshida et al., 2015). Recently it was shown that *Arabidopsis* plants overexpressing wheat transcription factor *TaAREB3* have enhanced ABA sensitivity and drought tolerance (Wang et al., 2016a) (**Table 1**). In primary species, group A PP2Cs have evolved as essential regulators providing osmotic stress tolerance (Komatsu et al., 2013).

**Table 1:** Major families of TF genes expressed in response to drought stress in plants.

Gene Family	Gene (Gene ID)	Identified in crop	Studied crop	Drought/other stress related function	References
*AREB/ABF*	*AREB1, AREB2/ABF4* and *ABF3*	*Arabidopsis thaliana*	*Arabidopsis thaliana*	Dehydration/Drought	Fujita et al., 2005; Yoshida et al., 2015
	*AREB1, AREB2/ABF4* and *ABF3*	*Arabidopsis thaliana*	*Arabidopsis thaliana*	Osmotic stress	Choi et al., 2000; Kang et al., 2002
					Kobayashi et al., 2008; Lee et al., 2010
	*ABF2*	*Arabidopsis thaliana*	*Arabidopsis thaliana*	Osmotic stress	Kim et al., 2004

*AP2/ERF*	*SodERF3*	*Saccharum officinarum L. cv Ja60-5*	*Nicotiana tabacum L. cv. SR1*	Dehydration/Multiple stress	Trujillo et al., 2008
	*GmERF3*	*Glycine max*	*Nicotiana tabacum*	Dehydration/Multiple stress	Zhang et al., 2009
	*AtDREB1A*	*Arabidopsis thaliana*	*Glycine max*	Dehydration	de Paiva Rolla et al., 2014
	*OsDREB1F*	*Oryza sativa*	*Oryza sativa*	Drought/Multiple stress	Wang et al., 2008
	*GmDREB2A;2*	*Glycine max*	*Glycine max*	Dehydration/Multiple stress response	Mizoi et al., 2013
	*DREB1/CBF*	*Arabidopsis thaliana*	*Oryza sativa*	Dehydration	Paul et al., 2015
	*FeDREB1*	*Fagopyrum esculentum*	*Arabidopsis thaliana*	Drought/Multiple stress	Fang et al., 2015
	*VrDREB2A*	*Vigna radiata*	*Arabidopsis thaliana*	Drought	Chen et al., 2016
	*EaDREB2*	*Erianthus arundinaceus*	*Saccharum* spp. *hybrid Co 86032*	Drought	Augustine et al., 2015
	*AtDREB2A*	*Arabidopsis thaliana*	*Glycine max*	Drought	Engels et al., 2013
	*OsDREB2A*	*Oryza sativa*	*Oryza sativa*	Drought	Cui et al., 2011
	*SsDREB*	*Suaeda salsa*	*Nicotiana tabacum*	Drought	Zhang X. et al., 2015
	*TaDREB2* and *TaDREB3*	*Triticum aestivum*	*Triticum aestivum, Hordeum vulgare L. cv. Golden Promise*	Drought	Morran et al., 2011

*NAC*	*SNAC1*	*Oryza sativa*	*Oryza sativa*	Drought	Hu et al., 2006
	*TaNAC2*	*Triticum aestivum*	*Arabidopsis thaliana*	Drought/Multiple stress	Mao et al., 2012
	*OsNAC5*	*Oryza sativa*	*Oryza sativa*	Drought	Song et al., 2011
	*OsNAC6*	*Oryza sativa*	*Oryza sativa*	Dehydration/Multiple stress	Nakashima et al., 2007
	*GmNAC20*	*Glycine max*	*Glycine max*	Drought	Hao et al., 2011
	*TaNAC69*	*Triticum aestivum*	*Triticum aestivum*	Dehydration Tolerance	Xue et al., 2011

*bZIP*	*GmbZIP44, GmbZIP62, GmbZIP78, GmbZIP132*	*Glycine max*	*Arabidopsis thaliana*	Salt and Freezing stress	Liao et al., 2008
	*GmbZIP1*	*Glycine max*	*Arabidopsis thaliana*	Drought	Gao et al., 2011
	*OsbZIP23*	*Oryza sativa*	*Oryza sativa*	Salinity and Drought tolerance	Xiang et al., 2008
	*Wlib19*	*Triticum aestivum*	*Nicotiana tabacum*	Abiotic stresses	Kobayashi et al., 2008
	*ZnbZIP17*	*Zea mays*	*Zea mays*	Drought, Heat, Salt	Jia et al., 2009
	*OsAREB1*	*Oryza sativa*	*Arabidopsis thaliana*	Drought, Heat	Jin et al., 2010
	*SlAREB*	*Solanum lycopersicum*	*Solanum lycopersicum, Arabidopsis thaliana*	Water deficit and Salt stress	Hsieh et al., 2010
	*OsbZIP16*	*Oryza sativa*	*Oryza sativa*	Drought	Chen et al., 2012
	*DgZFP*	*Dendronthema grandiform*	*Nicotiana tabacum*	Drought, Salinity, Cold	Liu et al., 2010
	*HvDRF1*	*Hordeum vulgare*	*Hordeum vulgare*	Drought	Xue and Loveridge, 2004

*Homeodomain-leucine zipper (HD-Zip) proteins*	*Multiple HD-Zip genes*	*Glycine max*	*Glycine max*	Drought/Salinity	Chen et al., 2014

*Zinc finger proteins*	*OsTZF1*	*Oryza sativa*	*Oryza sativa*	RNA metabolism of stress-responsive genes/Multiple stress response	Jan et al., 2013
	*DgZFP2*	*Dendronthema grandiform*	*Dendronthema grandiform*	Dehydration/Multiple stress	Liu et al., 2012
	*TaMYB33*	*Triticum aestivum*	*Arabidopsis thaliana*	Drought/Salinity	Qin et al., 2012
	*TaPIMP1*	*Triticum aestivum*	*Nicotiana tabacum*	Drought/Salinity	Liu et al., 2011


### AP2/ERF TFs

APETALA2/Ethylene Response Element binding Factors (*AP2/ERF*) family covers a large group of plant-specific TFs and is characterized by the presence of a much-conserved AP2/ERF DNA-binding domain (Song et al., 2013). This domain binds with the GCC box, which is a DNA sequence having role in the ethylene-responsive transcription (Rashid et al., 2012). *AP2/ERFBP* TFs perform diverse roles in plant biological processes, such as cell proliferation, vegetative and reproductive development, plant hormone responses, and abiotic/biotic stress responses (Sharoni et al., 2011; Xu et al., 2011). Genome-wide analyses have resulted in the identification of a multitude of *AP2/ERFBP* members across several plant species such as 145 in *Arabidopsis* (Riechmann and Meyerowitz, 1998), 170 in rice (Rashid et al., 2012), 178 in sorghum (Srivastav et al., 2010), 200 in poplar (Zhuang et al., 2008), 291 in Chinese cabbage (Song et al., 2013), 171 in foxtail millet (Lata et al., 2014), and 116 in moso bamboo (Wu et al., 2015). Based on the number and similarity of AP2/ERF domains, the family is further categorized into four major subfamilies: *AP2* (Apetala 2), *RAV* (related to ABI3/VP1), *DREB* (dehydration-responsive element-binding protein), and *ERF* (Sharoni et al., 2011; Rashid et al., 2012). Apropos of the plant abiotic and biotic stress response, *ERF* and *DREB* subfamilies have been extensively studied. Induced, respectively, by cold and dehydration, *DREB1/CBF* (with 11 genes in rice) and *DREB2* (six genes in rice) function in ABA-independent manner (Srivastav et al., 2010; Nakashima et al., 2014b). The promoter analysis of drought stress regulated genes having ABA-independent expression in *Arabidopsis* illustrated a *cis*-element with A/GCCGAC sequence (known as DRE/CRT: Lucas et al., 2011). Functional similarities were observed in the downstream genes of *DREB1A* and *DREB2A* by microarray analysis, however, differential expression was established during carbohydrate metabolism in transgenic plants. Four orthologs of *CBF/DREB1A* in rice *viz*. *OsDREB1A, OsDREB1B, OsDREB1C*, and *OsDREB1D* have already been characterized (Dubouzet et al., 2003). *DREB1/CBF* TFs specifically interact with the *DRE/CRT* and regulate the expression of several abiotic stress-responsive genes. Different studies confirmed that *DREB1/CBF* genes expression is differentially regulated by other genes like *ICE1, HOS1, MYB15, SIZ1, PIF7, CAMTA3*, and a clock component (Dong et al., 2011; Qin et al., 2011). Plants overexpressing *DREB1/CBF* showed enhanced expression of several stress responsive genes providing improved tolerance toward drought including tomato, chrysanthemum, potato (Iwaki et al., 2013), soybean (de Paiva Rolla et al., 2014), rice (Datta et al., 2012; Nakashima et al., 2014b; Paul et al., 2015), tobacco (Phuong et al., 2015), sugarcane (Augustine et al., 2015), groundnut (Tiwari et al., 2015), peanut (Bhatnagar-Mathur et al., 2014), and wheat (Shavrukov et al., 2016). In rice, *DREB1/CBF*-type TFs conferred higher drought tolerance besides expression of cold-responsive genes. Ishizaki et al. (2013) experimentally demonstrated improved survival of NERICA1 (an upland rice cultivar) under drought stress through expressing *Arabidopsis DREB1C* in the transgenic plants. *OsDREB1F* is reported to be induced by drought stress and ABA application (Wang et al., 2008). Overexpression of *OsDREB1G* is also known to promote drought tolerance (Chen et al., 2008). Similarly, constitutive expression of *DREB1/CBF3* in transgenic plants led to improved tolerance against salinity, heat and cold (Augustine et al., 2015; Fang et al., 2015; Kidokoro et al., 2015; Chen et al., 2016). Higher accumulation of osmoprotectants, such as free proline and soluble sugars were recorded in the transgenic rice plants overexpressing *DREB1A* (Ito et al., 2006). More recently, transgenic tobacco with elevated tolerance level against drought and salt was obtained through over-expression of *SsCBF4* from succulent halophyte *Suaeda salsa* (Zhang X. et al., 2015) (**Table 1**). An affymetrix microarray based analysis of transgenic rice plants facilitated the identification of 404 genes as induced or suppressed by *DREB1BI* when compared to their respective wild types (Zhuang et al., 2015).

A conserved regulatory mechanism has been established after examining DREB2-type proteins across several crop species such as wheat, maize, rice, barley, and sunflower (Mizoi et al., 2012). Rice harbors six *DREB2* family genes (Srivastav et al., 2010) while *Arabidopsis* contains eight *DREB2* genes of which *DREB2A* and *DREB2B* rendered higher induction under drought stress (Nakashima et al., 2014b). The constitutive as well as stress inducible expression of *OsDREB2A* and *OsDREB2B* in transgenic plants has resulted in enhanced tolerance toward osmotic stress (Cui et al., 2011; Mizoi et al., 2013). Transgenic *Arabidopsis* plants overexpressing *OsDREB2B* displayed enhanced expression of *DREB2A* target genes and improved tolerance toward drought stress (Matsukura et al., 2010). Taken together, these reports suggest that *OsDREB2* is a major gene encoding for *DREB2*-type TF that holds great importance in stress-responsive gene expression. However, in addition to imparting increased drought tolerance, over expression of *DREB2A* was reported to cause significant growth defects. In contrast, no growth defects were seen in case of *Arabidopsis* and soybean transgenics where stress-inducible expression of *DREB2A* led to enhanced drought tolerance (Engels et al., 2013). Under normal condition, *DREB2A* is regulated by two mechanisms (i) *GRF7* (growth-regulating factor7) inhibits expression of *DREB2A* binding to its short promoter region (Singh and Laxmi, 2015) and (ii) targeted degradation by 26S proteasome mediated proteolysis assisted by *DRIP1* (DREB2A- interacting protein1) and *DRIP2* protein (Morimoto et al., 2013; Singh and Laxmi, 2015). In other words, conditional expression of *DREB2A* under drought stress offers a way to minimize the inefficient loss of energy.

Besides *DREB1A* and *DREB2* members, various other *AP2/ERF*-type and *AP2/ERF*-like TFs were also characterized in rice with an aim to improve abiotic stress tolerance. Likewise, overexpression of *Arabidopsis HARDY* gene (a subgroup of A-4 AP2/ERF family) in *Trifolium* led to enhanced osmotic stress tolerance (Abogadallah et al., 2011). *OsDERF1* has been established as a negative modulator in osmotic adjustment by interacting directly with GCC box located in the promoter of *OsERF3* (Wan et al., 2011). Furthermore, *OsERF3* contains an ethylene-responsive element-binding factor-associated amphiphilic repression (EAR) motif, which transcriptionally represses the ethylene production along with drought tolerance. Mutant EAR lines showed enhanced ethylene emission and drought tolerance as compared to overexpressing as well as wild type plants (Zhang et al., 2013). However, Joo et al. (2013) reported *OsERF4a* as a positive regulator of plant growth and drought tolerance in rice. In addition, ectopic expression of tomato *JERF3* alleviates soluble sugars and proline with increased drought tolerance in rice (Zhang et al., 2010). Similarly, overexpression of another *AP2/ERF* TF showed increased drought, cold, and salinity stress tolerance in transgenic rice (**Table 1**).

### NAC TFs

The *NAC* gene family is the largest and plant specific TF family (Puranik et al., 2012; Shao et al., 2015). The NAC acronym is derived from three proteins having a specific NAC domain from petunia, i.e., *NAM* (no apical meristem) and *ATAF1/2* and *CUC2* (cup-shaped cotyledon) in *Arabidopsis* (Shao et al., 2015). Characteristically, a *NAC* TF comprises of a highly conserved DNA binding NAC domain in the N-terminal region and a variable C-terminal transcriptional regulatory region. The NAC domain is linked with DNA binding, nucleus-oriented localization and formation of homodimers or heterodimers with similar domains, while the C-terminal functions in transcriptional regulation (Puranik et al., 2012). The *NAC* TFs modulate downstream drought inducible EARLY RESPONSE TO DEHYDRATION1 (*ERD1*) gene transcription by interacting with NAC recognition sequence (NACRS) having CACG core-DNA binding motif in its promoter (Shao et al., 2015). In rice, *SNAC1* TFs were reported to confer drought tolerance through regulating downstream genes *OsPP18*, a PP2C in ABA independent pathways (You et al., 2014). To date, genome wide characterization has offered numerous putative *NAC* TFs across different plant species like 204 in Chinese cabbage, 152 in soybean, 152 in maize, 151 in rice, 117 in *Arabidopsis* and 74 in grape (Shao et al., 2015). In rice, several *NAC* genes were reported to be induced during early stages of salt and drought stresses (Hong et al., 2016). The role of *NAC* TFs in drought stress response is further supported by several transcriptome-based analyses undertaken in different crops; examples include 40 *NAC* genes in rice (Shao et al., 2015), 38 *NAC* genes in soybean (Le et al., 2011). These stress-responsive *NAC* genes also showed differential expression patterns such as tissue-specific, developmental stage-specific or stress-specific expression, thereby suggesting their active involvement in the complex signaling networks during plant stress responses. In *Arabidopsis*, drought tolerance was found to be elevated by overexpressing three genes, i.e., *ANACO19, ANACO55, ANACO72*. Similarly, overexpression of stress responsive *NAC1* (*SNAC1*) in rice provides tolerance against severe drought stress at the reproductive stage in field conditions without a change in their phenotype or yield penalty (Liu G. et al., 2014). In another study it was found that under drought stress *SNAC1* was induced in guard cells and its over-expression reduced transpirational losses due to increased stomatal closure (Singh et al., 2015b). Highlighting the importance of *SNAC1* gene, genetic variation of this gene was assessed among Indian wild rice and as a result, discovery of four haplotypes associated with tolerance might be useful for developing drought tolerant crops (Singh et al., 2015b). Similarly, *OsNAC5* expression was reported to be induced under drought, cold, ABA, and methyl jasmonate (MeJA) treatments. Overexpressors of *OsNAC5* were reported to serve as transcriptional activators that regulate the stress-responsive gene expression (Takasaki et al., 2010). Function of *OsNAC5* in drought tolerance was examined by RNA interference (RNAi) and overexpression study in transgenic rice and *Arabidopsis* (Song et al., 2011). In addition, *OsNAC6/SNAC2* is also known to be induced by cold, drought, salinity and ABA (Todaka et al., 2012). Furthermore, overexpressing *TaNAC2* and *TaNAC29* in *Arabidopsis* resulted in enhanced drought, salt and cold stress tolerance along with higher transcript levels of stress-responsive genes and improved physiological parameters (Mao et al., 2012; Huang et al., 2015). Contrary to this, *OsNAC6* overexpressing rice plants had reduced growth and low yield under normal conditions even though these plants remained tolerant for dehydration and salinity stresses. Based on these studies, *OsNAC6* could be established as a potent functional regulator in drought stress response (Todaka et al., 2012). Interestingly, overexpression of *HvSNAC1* in barley not only improved drought tolerance but also provided resistance against fungal infection of *Ramularia collo-cygni* causing leaf spot (Al-Abdallat et al., 2014; McGrann et al., 2015).

Enhanced abiotic stress tolerance in rice caused by overexpressing *SNACs* was evident when using a root-specific promoter RCc3 (Jeong et al., 2010, 2013; Redillas et al., 2012). Similarly, thicker roots and higher grain yield under drought stress were observed in transgenic rice plants overexpressing *OsNAC10* (Jeong et al., 2010), *OsNAC5* (Jeong et al., 2013), and *OsNAC9/SNAC1* (Redillas et al., 2012) regulated by root specific promoter (Jeong et al., 2010). Furthermore, microarray analysis of these transgenic plants revealed 62 downstream genes to be differentially expressed, which included *P450*, Zn-finger, *HAK5, 2OG-Fe(II), NCED, NAC, KUP3*, calcium-transporting ATPase, germin-like protein, and meristem protein (Redillas et al., 2012). Of these, only 17 were found to be upregulated (Jeong et al., 2013). However, as demonstrated by Hao et al. (2011) in soybean *GmNAC11* was reported to function as a transcriptional activator, whereas *GmNAC20* acted as a mild repressor with C-terminal transcriptional activation activity and its overexpression increases lateral root development in transgenic plants. This study established *GmNAC20* as a regulator of stress tolerance, which serves by activating DREB/CBF-COR pathway and lateral root formation by modulating auxin signaling genes (**Table 1**). Thus, precise manipulation of *SNAC*s with suitable promoter offers a promising way to substantially alter drought stress response in plants (Mei and Lizhong, 2011; Bang et al., 2013; Rusconi et al., 2013; Nakashima et al., 2014b).

### bZIP TFs

The basic leucine zipper (*bZIP*) family contains a conserved bZIP domain, which is composed of a highly basic nuclear localization and DNA binding region at the N-terminus and a leucine-rich motif for dimerization at the C-terminus (Wang et al., 2015). Besides regulating plant growth and development, *bZIP* TFs remain crucial concerning abiotic stress response such as drought (Llorca et al., 2014). Members of the *bZIP* TF family have been isolated and characterized in various eukaryotes. Examples include 17 in *Saccharomyces*, 31 in *Caenorhabditis*, 55 in grapevine (Liu J. et al., 2014), 75 in *Arabidopsis* (Jakoby et al., 2002), 89 in rice (Nijhawan et al., 2008), 89 in barley (Pourabed et al., 2015), 92 in sorghum (Wang et al., 2011), 96 in *Brachypodium distachyon* (Liu and Chu, 2015), 125 in maize (Wei et al., 2012), and 131 in soybean (Liao et al., 2008). Though the physiological role of their homologs with putative zinc finger motif remains unclear, studies on abiotic stress responses have confirmed *bZIP* TFs to be ABA inducible, i.e., these regulate the expression of stress-related genes in ABA-dependent manner after binding with the promoter region of specific ABRE (Llorca et al., 2014).

Xu et al. (2007) characterized a novel gene *ThZF1* from *Thellungiella halophila* that encodes a functional TF. It contains two conserved Cys-2/His-2 regions with conserved DNA-binding motif similar to the members of *Arabidopsis* ZFP family. Drought and salinity are reported to induce the transcription of this gene. Ectopic expression of *ThZF1* in *Arabidopsis* mutant *azf2* revealed its similarity with *Arabidopsis AZF2* in plant development and downstream gene regulation. Likewise, transgenic rice plants overexpressing *OsbZIP16* exhibited significantly higher drought tolerance at both seedling and tillering stages (Chen et al., 2012). Under drought stress conditions its downstream drought-inducible genes showed significantly higher expression levels in transgenics than the corresponding wild types. Though *OsbZIP16* is reported to be ABA-inducible, overexpression of *OsbZIP16* renders transgenic plants more sensitive to ABA. A recent study in *Arabidopsis* revealed that overexpression of *TabZIP60* improved plant’s tolerance against stresses like drought, salt and freezing along with increasing sensitivity to ABA (Zhang L. et al., 2015).

Similarly, *OsbZIP23*, a close relative of *Arabidopsis* homologs *ABF/AREB*, was reported to be a principle regulator of ABA-dependent pathways (Todaka et al., 2015). Rice plants overexpressing *OsbZIP23* caused greater ABA sensitivity at both germination and post-germination stages along with increased drought and salt stress tolerance. Microarray analysis showed differential expression of several downstream genes including stress-related TFs, protein kinases, dehydrins and LEA proteins. *OsbZIP46* also belong to the subfamily of *OsbZIP23* and its constitutively active form *OsbZIP46CA1* was developed by mutating *OsbZIP46* domain D. Rice plants overexpressing *OsbZIP46CA1* showed enhanced drought tolerance. Differentially expressed downstream genes different from those of *OsbZIP23* downstream genes were uncovered through a microarray analysis, which in turn suggested independent regulatory mechanisms of *OsbZIP46CA1* and *OsbZIP23*. In contrast, growth of *OsbZIP23* or *OsbZIP46CA1* overexpressing rice plants is reduced under drought stress (Tang et al., 2012). Furthermore, ABF3 belonging to similar subfamily as *OsbZIP46* and *OsbZIP23* improved drought tolerance in transgenic *Arabidopsis* and rice (Todaka et al., 2015). *OsbZIP16* and *OsbZIP71* are classified into group IV of the rice *bZIP* subfamily (Todaka et al., 2015). After ABA treatment transgenic rice plants overexpressing *OsbZIP16* showed enhanced drought tolerance and reduced plant growth (Chen et al., 2012). However, *OsbZIP71* overexpressing rice transgenics with a constitutive promoter or a stress-inducible RD29A promoter showed enhanced drought, salt, and osmotic stress tolerance (Liu J. et al., 2014). Collectively, these reports confirmed the involvement of *bZIP* TFs in ABA signaling, which could be harnessed for developing better genotypes endowed with drought tolerance (Todaka et al., 2015).

### Interactions among Multiple TFs

Drought stress is an unpredictable event and it varies in severity and duration. This results in both general and specific effects on plant growth and development. Thus, plant response toward drought stress is dynamic, which involves multiple stress perception and signal transduction pathways, which may crosstalk at various steps in the pathways (Lenka et al., 2011). For this reason, plants have evolved complex regulatory mehanisms, including metabolic adjustment and gene expression toward physiological and morphological adaptation (Baldoni et al., 2015). Moreover, extensive overlap between signal interactions and adaptation mechanisms can be both synergistic and antagonistic, resulting in positive and negative functional outcomes (Atkinson and Urwin, 2012). This type of regulation allows cell to respond rapidly to changes such as drought stress (Lindemose et al., 2013). TFs are of key importance in these signaling cascades and in generating specificity in stress responses. Interactions among different TFs have made significant advances over recent years and were documented recently by Nakashima et al. (2014a). The TFs control transcription of their downstream genes by interacting with other proteins and binding to a consensus sequence in promoters. Identification of these downstream genes becomes imperative to elucidate molecular machineries of gene activation or repression (**Figure 1**).

Drought responsive gene promoters contain a DRE/CRT motif where ABA-independent *DREB/CBF* TF binds and act as a coupling element for ABRE in ABA-dependent gene expression (Singh and Laxmi, 2015). It was already shown that *DREB1A/CBF3, DREB2A*, and *DREB2C* proteins interact with *AREB/ABF* (Lee et al., 2010). Thus, there exists a crosstalk between ABA-dependent and independent signaling and regulatory pathways (**Figure 1**). Under osmotic stress conditions, *AREB/ABF* TFs and *SnRK2*s regulate the transcriptional activation of *DREB2A* gene, suggesting a complex interaction between *DREB* and *AREB* regulatory regions at the transcript and protein level (Kim et al., 2011). Similar interactions were also reported in between *AREB/ABFs* and NACs. In addition, regulation of ABA-dependent gene expression of ABRE regulons by *SNAC* TFs was confirmed when Jensen et al. (2013) reported that *Arabidopsis SNAC* transcription factor *ATAF1* directly modulates ABA biosynthetic gene *NCED3*. By contrast, *SNAC* gene promoter contains ABRE region (Nakashima et al., 2014b). Under dehydration and osmotic stress, *ANAC096* was also found to interact with *ABF2/AREB1* and *ABF4/AREB2* (Xu et al., 2013). Furthermore, correlation among *DREB/CBF*s and *AP2/ERF*s at the transcript levels was also confirmed when *Arabidopsis ERF1* was shown to regulate transcript expression of a gene by coupling with two different *cis*-elements, the GCC box and DRE/CRT during stress response (Cheng et al., 2013). Recently, it was shown that *bZIP* TFs *VEG2* and *FLOWERING LOCUS D* (*FD*) interact with nuclear protein *CONSTANS* (*CO*) and positively regulate signaling of flowering activator *FLOWERING LOCUS T* (*FT*) proteins (Cai et al., 2014; Weller and Ortega, 2015). TFs activate several functional genes encoding wide range of proteins involved in osmolyte production, ROS scavenging and detoxification, macromolecule protection and ubiquitination. As envisaged by Puranik et al. (2012), such versatility in TFs functioning might have evolved to ensure plants’ longevity, survival and reproductive success under environmental stresses. Our current knowledge about these target genes stems mainly from the studies performed using large-scale transcriptome analyses in plants that overexpress TFs. Deeper analyses can better elucidate their roles under drought response.

### Future Directions

Here, we highlighted the increasingly important roles of TFs as modern genomic tools to improve plant tolerance toward multiple abiotic stresses. Significant progress has been achieved in deciphering the role of TFs toward tolerance to abiotic stress factors such as drought, and an appreciable number of promising candidates – TF genes have already been identified and validated. Selection of key TFs from such large families and realization of their full potential still remain a strenuous task. Also, development of transgenic plants harboring such TF genes that deliver anticipated level of tolerance in the field conditions still poses a great challenge to the research community. Furthermore, functional redundancy between different TF members might potentially hamper the progress to delineate the functions of an individual member.

The constitutive over-expression of some TF genes may improve the stress tolerance; however, occasional negative effects in transgenic plants such as dwarfing, late flowering and lower yields should also be taken into account while investing research. Furthermore, the complete regulatory mechanism of individual TF encompassing its upstream and downstream co-regulators, as well as their interactions remain largely obscure. A principle concern is also about the selection of plants, as most of the initial experiments are being performed on model crops like *Arabidopsis* and tobacco. Focus must be shifted to commercial crops like rice, wheat, maize and other cereal crops, which are predominantly affected by drought stress. In comparison to other crops, rice has shown the highest potential under waterlogging conditions (Voesenek and Bailey-Serres, 2013). Although reports are available with improved drought tolerance in transgenic rice under field conditions, further research is required in order to unveil the regulatory mechanism of drought tolerance. Future investigations must focus on pinpointing novel genes that enhance drought tolerance as well as yield. In addition, combination of drought-tolerance with submergence-tolerance in rice is exceptionally useful, as this would allow crop to withstand both drought and waterlogged conditions.

Another attractive approach for discovering novel candidate genes is to intensively examine the mechanisms that describe drought tolerance in extremophiles, i.e., desert plants (Mittler and Blumwald, 2010; Joshi et al., 2015). It is important to mention here that up to 38% of total protein functions are still unknown even in well-analyzed plants and a renewed focus in this aspect might led to the discovery of robust candidate genes. Modification in root architecture is another important factor for improving drought tolerant crops. Transcriptome analysis has shown that drought-responsive TFs like *REB1/CBF, DREB2, AREB/ABF*, and *NAC* TFs function under drought response and tolerance. Utilizing a balanced crosstalk between these TFs under osmotic stress, overexpression of TFs may affect their signaling pathways. Identification of multiple stress-responsive TF genes and their prioritization by means of comparing expression patterns deserves attention of the scientific community and pinpointing the commonly regulated genes which have been proposed to be essential for universal stress responses or represent points of cross-talk between signaling pathways (Prasch and Sonnewald, 2015). Genetic manipulation of these multiple stress-responsive TF genes stands to be a powerful approach for improving plant tolerance than addressing each functional gene individually. Furthermore, the field trials are required to critically evaluate the transgenic plants, especially focusing on their growth and tolerance in the whole life period, which unequivocally remains a deciding factor while developing stress-tolerant crops.

## Author Contributions

SLS-P and AP conceived the review topic. RJ, BS, and AB drafted the manuscript. SW, ZD, and AL participated in the discussion of topic. All authors listed, have made substantial, direct and intellectual contribution to the work, and approved it for publication.

## Conflict of Interest Statement

The authors declare that the research was conducted in the absence of any commercial or financial relationships that could be construed as a potential conflict of interest.
